# A Novel Humanized Anti-Interleukin-6 Antibody HZ0408b With Anti-Rheumatoid Arthritis Therapeutic Potential

**DOI:** 10.3389/fimmu.2021.816646

**Published:** 2022-01-19

**Authors:** Xiaolei Liu, Li Li, Qian Wang, Fengchao Jiang, Pei Zhang, Fei Guo, Hongjun Liu, Jian Huang

**Affiliations:** ^1^ Department of Medicine, Perelman School of Medicine at the University of Pennsylvania, Philadelphia, PA, United States; ^2^ IPHASE Therapeutic Ltd., Philadelphia, PA, United States; ^3^ National Health Commission (NHC) Key Laboratory of Systems Biology of Pathogens, Institute of Pathogen Biology, and Center for AIDS Research, Chinese Academy of Medical Sciences & Peking Union Medical College, Beijing, China; ^4^ Coriell Institute for Medical Research, Camden, NJ, United States; ^5^ Temple University Lewis Katz School of Medicine, Center for Metabolic Disease Research, Philadelphia, PA, United States; ^6^ Cooper Medical School of Rowan University, Camden, NJ, United States

**Keywords:** monoclonal antibody, rheumatoid arthritis, IL-6, autoimmune disease, collagen-induced arthritis (CIA) model

## Abstract

Interleukin-6 (IL-6), a pleiotropic cytokine that regulates immune responses and inflammatory reactions, plays a pivotal role in the development of rheumatoid arthritis (RA). Blockade of IL-6 signaling with the monoclonal antibody (mAb) represents an important advancement in RA treatment. Although two IL-6 receptor antibodies are already available in the clinic, there is no mAb specifically targeting the human IL-6 to block IL-6 signaling for RA treatment. In this study, we have developed a novel humanized anti-IL-6 mAb HZ-0408b with potent binding and neutralizing activity to human IL-6. We demonstrated that HZ-0408b has a high species specificity and low cross-reactivity. Moreover, HZ-0408b showed a more potent inhibitory effect on IL-6 signaling than Siltuximab, an FDA-approved anti-IL-6 chimeric mAb. HZ-0408b is comparable to Olokizumab, a humanized mAb against IL-6 that is already in phase III studies. We observed that HZ-0408b is well tolerated at doses that can achieve therapeutic serum levels in cynomolgus monkey. Most importantly, we proved that HZ-0408b treatment significantly ameliorated joint swelling after the onset of arthritis and dramatically reduced plasma C-reactive protein (CRP) levels in a monkey collagen-induced arthritis (CIA) model. Collectively, our findings using non-human primates indicate that humanized anti-IL-6 mAb HZ-0408b has excellent safety and efficacy profiles for RA therapy.

## Introduction

Rheumatoid arthritis (RA) is a chronic autoimmune disorder that is characterized by inflammation of synovial joint tissues and which affects up to 1% of the world’s population. In the past two decades, it became clear that abnormal activation of the complex network of proinflammatory cells and cytokines triggers the pathogenesis of RA. Among the factors involved in RA, interleukin (IL)-6 plays an essential role in the chronic inflammation associated with RA.

IL-6 is a pleiotropic cytokine with a wide range of biological activities in immune regulation, inflammation, hematopoiesis, and oncogenesis (1, 2). Since the discovery of IL-6 in 1986 (3), remarkable progress has been made in the understanding of the IL-6 receptor system, signaling transduction mechanism, and its biological activities. IL-6 exerts its biological activities through two receptor components: an 80-kDa ligand-binding chain (IL-6R) and a 130-kDa non-ligand-binding signal transducer glycoprotein 130 (gp130) (4). IL-6 stimulation induces tyrosine phosphorylation and recruitment of transcriptional factor signal transducer and activator of transcription 3 (STAT3), which dimerizes and is translocated to the nucleus to initiate the transcription of specific genes (5).

In RA, overproduction of IL-6 in the synovium results in the development of chronic synovitis and the proliferation of fibroblast-like synoviocytes, promoting angiogenesis and cartilage degradation in the synovium (6). Beyond the joint, IL-6 overproduction is also involved in extra-articular manifestations and common comorbidities in patients with RA, such as cardiovascular diseases, osteoporosis, and depression (6, 7). IL-6 inhibition targeting IL-6R has shown beneficial effects not only within the joint, but also in the extra-articular manifestations of RA in several clinical studies (8, 9). To date, two agents (Tocilizumab and Sarilumab) targeting IL-6R are clinically available for treating autoimmune diseases, including RA. For example, Tocilizumab, a humanized monoclonal antibody (mAb), was first approved in 2005 in Japan for Castleman’s disease (10). Subsequently, Tocilizumab was approved by FDA in 2010 for various indications, including moderately to severely active RA in adults who have had an inadequate response to one or more disease-modifying anti-rheumatic drugs (DMARDs) (11). In contrast to the impressive results for the IL-6R inhibitors, the development of agents targeting IL-6 for RA treatment remains stalled. For example, Sirukumab, the most advanced anti-IL-6 ligand monoclonal antibody, completed phase III trials but was rejected for approval for the treatment of active RA by the US FDA in August 2017 due to safety concerns. Olokizumab, another mAb against IL-6 for RA treatment, demonstrated beneficial effects in phase II trials, is still in phase III trials (12). Thus, a new efficacious agent targeting IL-6 is urgently needed for RA therapy.

In this study, we report that a novel humanized anti-IL-6 mAb HZ-0408b selectively binds to the IL-6 cytokine with high affinity and specificity. Furthermore, HZ-0408b is well tolerated at doses that can achieve potentially therapeutic serum levels in cynomolgus monkey, and it can ameliorate established arthritis in collagen-induced arthritis (CIA) cynomolgus monkey.

## Materials and Methods

### Generation and Humanization of HZ-0408b

HZ0408b (iPhase Bioscience, #062A01.11) is a genetically engineered monoclonal antibody, humanized from a mouse antihuman IL-6 antibody using a complementarity-determining region (CDR) grafting method. Hybridomas were generated using standard protocols. In brief, Kunming (KM) mice were immunized with purified recombinant IL-6His fusion protein. Titers were then assessed, and the spleen cells were fused with SP2/0 cells. Hybridomas were selected, and supernatants from the resulting clones were screened by the enzyme-linked immunosorbent assay (ELISA). Anti-IL-6 antibodies were further characterized for their ability to inhibit IL-6 induced STAT3 phosphorylation in the DLD-1 cell line (ATCC #CCL-221). One of the positive clones was obtained and its variable regions were cloned onto a human full-length IgG framework and subsequently humanized and engineered to minimize interactions with the immune system. Humanization was performed by grafting the CDRs into the closest human variable (V) region light- and heavy-chain framework sequences as previously described (13, 14). ELISA assay and inhibition of STAT3 phosphorylation were used to monitor the potency of humanized variants.

### Cell Culture

DS-1 cells (ATCC #CRL-11102) and DLD-1 cells (ATCC #CCL-221) were cultured in RPMI1640 (ATCC #30-2001) supplemented with 10% Fetal Bovine Serum (Hyclone #SH30071.03) and 1% penicillin/streptomycin (GIBCO #15140) and were maintained at 37°C and 5% CO2. Hep G2 cells (ATCC #HB-8065) were cultured in EMEM (ATCC #30-2003) supplemented with 10% Fetal Bovine Serum (Hyclone #SH30071.03).

### Cell Surface Antigen-Binding Assay

Binding activity to IL-6 and inhibitory activity of IL-6 binding to IL-6R were measured by ELISA as previously described (15). Briefly, 96-well plates were coated with 1ug/ml rhIL-6-His fusion protein (iPhase Bioscience, #051A03.11) in phosphate-buffered saline (PBS) overnight at 4°C. After blocking for 1 hr with 0.4% BSA in PBS at room temperature, increasing concentrations of humanized anti-IL-6 mAbs and Siltuximab were added to the plates at room temperature for 2 hr. For IL-6 and IL-6R interaction blocking assay, 96-well plates were coated with 1.5 μg/ml of IL-6R fusion protein (iPhase Bioscience, #051A09.21) in PBS for 16 hr at 4°C. 1μg/ml of IL-6-His protein was added either in the absence or presence of increasing concentrations of humanized anti-IL-6 mAbs and Siltuximab at room temperature for 2 hr. Plates were subsequently washed three times and incubated with an HRP-conjugated anti-His secondary antibody for 1 hr at room temperature. After washing, plates were developed with TMB. The OD was measured at 450 nM. Each sample was tested in triplicates. Competition ELISA of HZ-0408b and Siltuximab binding to rhIL-6-His were analyzed by competitions with equal amounts of limiting diluted concentrations of HZ-0408b and Siltuximab at 100ug/ml, 25ug/ml, and 6.26ug/ml, respectively.

### Binding Affinity

The binding affinity of humanized anti-IL6 antibody was measured against IL-6 using Bio-layer Interferometry (BLI) by FroteBio Blitz (Pall, USA). Anti-human Fc Capture (AHC) biosensors (Cat No, 18-5060, Fortebio, Pall) were used to probe purified antibodies at concentrations from 100 to 400 nM. The association and disassociation kinetics of binding to rhIL-6-His was measured using the following setting: initial baseline for 30 secs, followed by loading of antibodies for 300 secs, baseline for 60 secs, association for antigen for 300 secs and dissociation for 300 secs. The binding data were globally fitted to a 1:1 binding model to calculate the equilibrium dissociation constant (KD), association constant (Ka), and dissociation constant (Kd).

### Western Blot

Cells were lysed in buffer containing 20mM Tris pH 7.5, 140mM NaCl, 1mM EDTA, 10% glycerol, 1% Triton X-100, 1mM DTT, 50mM NaF, and protease inhibitor cocktail (Sigma P8340), phosphatase inhibitor cocktail #1 (Sigma P2850) and #2 (Sigma P5726) or #2 and #3 (Sigma P0044) used 1:100 each. Supernatants were collected after centrifugation at 13,000 rpm for 5 min at 4°C, adjusted to 1x laemmli sample buffer, and subjected to SDS-PAGE and then immunoblotted as described previously. To determine the type of epitope for HZ-0408b, 30ug heated rhIL-6-his was subjected to SDS-PAGE and HZ-0408b was used as the primary antibody for immunoblot assay.

### Animals

Male and female cynomolgus monkeys weighing 2-4 kg, age 3-6 years old were purchased from Beijing Prima BioTech Inc. All procedures involving animals were approved by the animal care and use committee of the laboratory and were performed in accordance with standards published by the National Research Council (Guide for the Care and Use of Laboratory Animals, NIH OACU), the National Institutes of Health Policy on Human Care and Use of Laboratory Animals. The monkeys were housed in cages and maintained on a 12 h light/12 h dark cycle.

### Toxicity and Pharmacokinetic Analysis in Cynomolgus Monkey

All animals were monitored twice daily for mortality and moribundity checks. Body weights were measured at least once pre-study and weekly thereafter starting on day 7. Blood was collected by venipuncture into tubes with no anticoagulant at different time points as indicated in **Figure S3**. Serum level of HZ-0408b was measured by ELISA as described above using IL-6 protein as the coating reagent, followed by detection with an HRP-conjugated anti-human IgG secondary antibody.

### Induction of Collagen-Induced Arthritis

The monkey CIA was induced by modifying the method that was reported previously (16). Briefly, four 5-6 years old female cynomolgus monkeys were used to generate the CIA model. Bovine type II collagen (4 mg/ml in Ethanoic acid, Chondrex, Cat#20021) was emulsified in Freund’s Complete Adjuvant (FCA, Chondrex, Cat# 7008). The prepared emulsion was maintained on ice until use. Each monkey was administered intradermally 2ml of prepared emulsion dorsally. Three weeks later, a second sensitization was performed in the same manner. We used the body weight, joint swelling (PIP), and serum CRP levels to determine the development of arthritis.

### Measurement of Joint Swelling

The longitudinal and transverse axes of PIP joints of the fore and hind limbs (without thumb) were measured with calipers, and the oval area of each PIP was calculated. The mean oval area of 16 PIP joints was calculated and adopted as individual data. The percent of oval area and joint swelling was calculated as previously described [11].

### Blood Chemistry

Plasma was obtained by centrifugation immediately after collection. Blood chemical parameters were measured by using an automatic chemistry analyzer (Mindray, BS220). Blood chemical parameters were as follows: C-reactive protein (CRP), aspartate aminotransferase (AST), alanine aminotransferase (ALT), total cholesterol (TC), lactate dehydrogenase (LDH), and triglyceride (TG). Plasma concentrations of IL-6 and soluble IL-6R (sIL-6R) were measured by ELISA kit for human IL-6 (R&D, Cat# DY206-05) and human IL-6R (R&D, Cat# DY227), respectively.

### Statistical Analysis

For pharmacokinetic (PK) analysis, DAS2.0 was used to calculate AUC (0-∞), AUC(0-t), T_max_, C_max_, t1/2, Vz, and CL. Calculation of relative potencies of the antibodies was performed in GraphPad Prism 8 software with a sigmoidal dose-response model. All data are presented as mean ± SD.

## Results

### Generation of Monoclonal Antibodies Against Human IL-6

First, we fused a cDNA fragment of human IL-6 to 6His-tag to generate 6His-IL-6 fusion protein, which was subsequently used to immunize mice to produce monoclonal mouse anti-human IL-6 antibody. Then we examined the specificity of selected hybridoma clones by ELISA to evaluate the antigen-binding specificity of the anti-IL-6 antibodies. To assess whether the anti-IL-6 antibody neutralized IL-6 activity, we tested the inhibitory effect of anti-IL-6 antibodies to IL-6 induced JAK-STAT3 signal activation by measuring protein level of p-STAT3 (Try705) in DLD-1 cells with IL-6 pre-treatment (Data not shown). Based on the result, we selected one of the best positive clones and designated it as #140-4.

Using the universal antibody primers, we successfully cloned heavy- and light-chain variable regions of #140-4 and determined the amino acid sequences of five VL (1K, 3K, 5K, 6K, 7K) and two VH (2H, 4H). We then cloned the variable sequences into the plasmid pcDNA3.1, which contains human CL-IgK and CH-IgG1 sequences. Next, we tested the binding activity of IL-6 antibodies to IL-6 by ELISA assay and measured p-STAT3 levels by western blot. Our results showed that the combination of VL-3K and VH-4H had the highest antigen-binding affinity and neutralizing activity to recombinant IL-6 (Data not shown).

### Humanization, and Characterization of the Antibody

To humanize the antibody, we selected the variable regions of light-chain (3K) and heavy-chain (4H) and humanized the antibody using framework regions (FRs) of the closest human germline sequences. Next, we assembled humanized VL-3K (3K-1, 3K-2, and 3K-3) and VH-4H (4H-1, 4H-2, and 4H-3) into pcDNA3.1 plasmid containing human IgK or IgG1 constant regions. Then we generated humanized anti-IL-6 antibodies by co-transfecting the paired plasmids into HEK293T cells followed by protein A purification.

**Figure 1:**
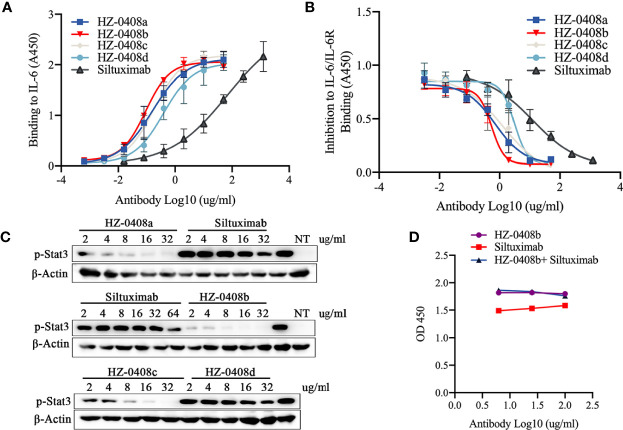
Generation, humanization, and characterization of anti-human IL-6 monoclonal antibody HZ-0408b. **(A)** The binding activity of the humanized anti-IL-6 mAbs and Siltuximab to IL-6 was measured by ELISA using an HRP-conjugated anti-human IgG secondary antibody. Each sample was assayed in triplicates. Mean absorbance at 450nm and SD values are presented. **(B)** The inhibitory effect of humanized anti-IL-6 mAbs and Siltuximab on IL-6 and IL-6R binding was measured by ELISA. Each sample was performed in triplicates. Mean absorbance at 450nm and SD values are presented. **(C)** The humanized anti-IL-6 mAbs and Siltuximab inhibited STAT3 signaling activity. DLD-1 cells were cultured in the presence of 10ng/ml IL-6 and indicated concentrations of humanized anti-IL-6 mAbs and Siltuximab were added for 2 hr. STAT3 phosphorylation at T705 was measured by western blot. NT, nontreated with IL-6. **(D)** HZ-0408b and Siltuximab binding to rhIL-6-His were analyzed by competitions with equal amount of limiting diluted HZ-0408b and Siltuximab at 100ug/ml, 25ug/ml, and 6.26ug/ml, respectively as described in Methods.

To assess the antigen-binding activity of the humanized anti-IL-6 mAb to IL-6, we performed ELISA using the rhIL-6-His coated plates as described before. We used Siltuximab — an FDA-approved anti-IL-6 mAb under the brand name of Sylvant for the treatment of patients with idiopathic multicentric Castleman’s disease (iMAD) — as a control (**Figure 1A**). All of our humanized IL-6 antibodies (HZ-0408a, HZ-0408b, HZ-0408c, and HZ-0408d) are effective on binding to rhIL-6-His in a dose-dependent manner, and our antibodies showed significantly higher binding activity to IL-6 than Siltuximab.

Furthermore, we measured the binding affinity of the humanized anti-IL6 antibody against IL-6 using Bio-layer Interferometry (BLI). The equilibrium dissociation constant (KD) of HZ0408b for IL-6 was 1.075e-9 M, which is ten times lower than Siltuximab (1.168e-8 M), a result indicating that the affinity of HZ-0408 to rhIL-6 is dramatically higher than Siltuximab. Importantly, we found that the difference was derived from a higher association constant (Ka) of HZ-0408b (2.333e5 1/Ms) than that of Siltuximab (2.052 e4 1/Ms), whereas the dissociation constant (Kd) was similar between two antibodies (2.507e-4 1/s of HZ-0408b vs 2.396e-4 1/s of Siltuximab). These results suggest that HZ-0408b has a similar dissociation rate but stronger binding activity to IL-6 when compared with Siltuximab.

We then tested the ability of the humanized mAbs to block the interaction between IL-6 and IL-6R. We coated 96-well plates with recombinant IL-6R and measured the binding of the 6His-IL-6 fusion protein. As shown in **Figure 1B**, IL-6 bound IL-6R without the presence of the antibodies; however, the binding activity was blocked in a dose-dependent manner when the humanized anti-IL-6 mAbs were added. Complete inhibition was achieved at 10ug/ml of our humanized mAbs — much lower than that of Siltuximab.

To test the neutralizing activity of the humanized antibody, we measured the levels of STAT3 signaling activity in DLD-1 cells with IL-6 pre-treatment. As shown in **Figure 1C**, our humanized anti-IL-6 antibodies HZ-0408a, HZ-0408b, HZ-0408c, HZ-0408d, as well as Siltuximab, can inhibit STAT3 signaling activity in a dose-dependent manner. The levels of p-STAT3 are significantly lower in the humanized anti-IL-6 mAbs treated groups compared to Siltuximab, and HZ-0408b is the most potent antibody in inhibiting STAT3 activity.

We further tested the species specificity of the antibody and found that HZ-0408b does not bind to mouse, rat, or rhesus monkey IL-6 (Data not shown). We further tested the cross-reactivity of HZ-0408b to other cytokines. ELISA results demonstrate that HZ-0408b does not have a binding activity to other IL-6 family cytokines such as IL-11, oncostatin M (OSM), leukemia inhibitory factor (LIF), and others (**Figure S1**). Because gp130 is a receptor component that is shared with other cytokines of the IL-6 family, we also tested whether HZ-0408b binds to gp130. As shown in **Figure S1**, HZ-0408b does not bind to gp130, which suggests that HZ-0408b is highly specific in inhibiting IL-6 signaling.

We then performed an immunoblot assay to determine the type of epitope. Briefly, we used the heat-denatured rh-IL-6-his protein for SDS-PAGE immunoblot assay and HZ-0408b as the primary antibody. The results showed that HZ-0408b recognized the linear epitope of IL-6 (**Figure S2**). We then performed competition ELISA with HZ-0408b and Siltuximab. We coated the 96-well plate with hIL-6-his and then added limiting diluted HZ0408b with or without an equal amount of Siltuximab. Our results showed that the two antibodies recognized the similar or same epitope (**Figure 1D**). Overall, these results suggest that HZ-0408b has potent binding and neutralizing activity to human IL-6 with high species specificity and low cross-reactivity.

### The Humanized Anti-IL-6 Antibody Can Inhibit IL-6 Induced Serum Amyloid A Secretion and Cell Proliferation

**Figure 2:**
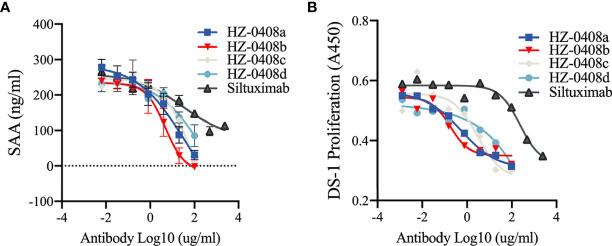
The humanized anti-IL-6 mAbs are effective on inhibiting IL-6 induced SAA secretion and IL-6 dependent cell proliferation. **(A)** The humanized anti-IL-6 mAbs and Siltuximab inhibited the induction of SAA expression by IL-6 and IL-1β in an does-dependent manner in HepG2 cells. HepG2 cells (2X10^5^) were plated in 48-well plate and incubated with serially diluted humanized anti-IL-6 mAbs or Siltuximab in the presence of 100 ng/ml IL-6, 200 ng/ml IL-6R, and 25 ng/ml IL-1 for 48 hr. Supernatant was collected for ELISA. **(B)** *In vitro* growth-inhibitory effect of the humanized anti-IL-6 mAbs and Siltuximab in IL-6-driven expansion of DS-1 cells. DS-1 cells (8×104/mL) were incubated with 20 ng/ml IL-6 in the presence of serially diluted humanized anti-IL-6 mAbs or Siltuximab for 72 hr. Cell counting kit-8 (CCK8) was used to measure the cell proliferation, and absorbance at 450nm are presented.

We then examined whether the humanized anti-IL-6 mAb can inhibit IL-6 stimulated secretion of serum amyloid A (SAA), which is mainly produced in the liver and dramatically increased during inflammation. SAA is a precursor of amyloid A (AA) protein in AA (secondary) amyloidosis, which is a serious complication of chronic inflammatory diseases, including RA (17). We found that SAA secretion could be induced with the presence of rhIL-6-his (100ng/ml) and IL-1β (25ng/ml) in the hepatic cell line HepG2 and that IL-6 blockade with our humanized anti-IL-6 monoclonal antibodies HZ-0408a (IC_50_ = 3.695ug/ml), HZ-0408b (IC_50_ = 2.481ug/ml), HZ-0408c (IC_50_ = 17.79ug/ml), HZ-0408d (IC_50_ = 12.09ug/ml), as well as Siltuximab (IC_50_ = 18.42ug/ml), inhibits the synergistic induction of SAA expression by IL-6 and IL-1β in a dose-dependent manner (**Figure 2A**). Compared to Siltuximab, all of our humanized anti-IL-6 mAbs are significantly superior in inhibiting SAA secretion.

Moreover, exposure to the humanized anti-IL-6 monoclonal antibodies also diminished IL-6-driven expansion of DS-1, a B-lymphoma cell line, whose continuous growth is supported by endogenous IL-6 and is enhanced by exogenous IL-6. As shown in **Figure 2B**, in the presence of IL-6 (20ng/ml), addition of the anti-IL-6 mAbs suppressed growth of IL-6-dependent cell line DS-1, an effect that was also dose-dependent. IC-50 of our humanized anti-IL-6 mAbs is significantly lower than Siltuximab. These results demonstrated that our novel humanized anti-IL-6 mAbs are more potent in inhibiting IL-6 signaling activity than Siltuximab.

### HZ-0408b Is Comparable to Olokizumab in Blocking IL-6 Signaling

**Figure 3:**
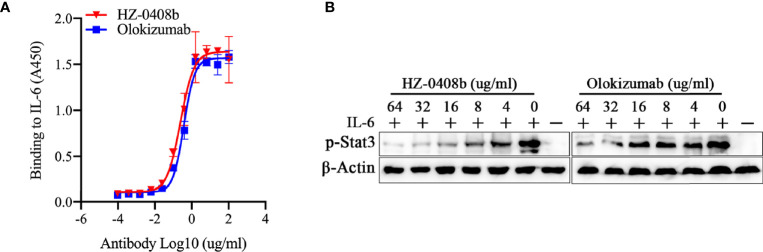
HZ-0408b is comparable to Olokizumab in blocking IL-6 signaling. **(A)** The binding activity of the HZ-0408b and Olokizumab to IL-6 was measured by ELISA using an HRP-conjugated anti-human IgG secondary antibody. Each sample was assayed in triplicates. Mean absorbance at 450nm and SD values are presented. **(B)** HZ-0408b and Olokizumab inhibited STAT3 signaling activity. DLD-1 cells were cultured in the presence of 10ng/ml IL-6 and indicated concentrations of HZ-0408b and Olokizumab were added for 2 hr. STAT3 phosphorylation at T705 was measured by western blot.

We further compared the antigen-binding activity and neutralizing activity of HZ-0408b to Olokizumab, which is a humanized mAb against IL-6 for RA treatment and already in phase III studies [10]. We found that HZ-0408b has comparable antigen-binding activity to Olokizumab by ELISA (**Figure 3A**). Using BLI assay for binding affinity test, we found that the KD of HZ0408b for IL-6 was 3.474e-8 M, while Olokizumab was 2.425e-8 M, which indicates that the binding affinity of HZ-0408 to rhIL-6 is similar to Olokizumab. To compare the neutralizing activity of HZ-0408b to Olokizumab, we measured STAT3 signaling activity in DLD-1 cells with IL-6 pre-treatment. Both HZ-0408b and Olokizumba can inhibit STAT3 signaling activity in a dose-dependent manner, as determined by decreased p-STAT3 levels (**Figure 3B**). Of note, HZ-0408b showed slightly higher efficacy in inhibiting STAT3 signaling activity compared to Olokizumba (**Figure 3B**). These results demonstrated that HZ-0408b was comparable to Olokizumab in blocking IL-6 signaling.

### The Humanized Anti-IL-6 Antibody Demonstrated Safety in Non-Human Primate Toxicology and Pharmacokinetic (PK) Studies

For toxicology assessment, we tested HZ-0408b administered to cynomolgus monkey as a single subcutaneous injection at 1 and 5 mg/kg in separate individuals. All animals were evaluated for changes in clinical signs, food consumption, body weights, and clinical pathology parameters. No treatment-related effects were observed, which suggests that administration of HZ-0408b was well tolerated in non-human primates.

To characterize the PK parameters of HZ-0408b, we dosed both male and female cynomolgus monkeys with increasing amounts of HZ-0408b (1mg/kg, and 5mg/kg) and collected blood samples both before dosing and several time points post-dosing for analysis of HZ-0408b in plasma. With single-dose administration of HZ-0408b, PK data demonstrated that both 1 and 5 mg/kg dose levels were able to transiently achieve detectable serum levels (**Figure S3**). The low doses of HZ-0408b were rapidly cleared from serum. In contrast, the higher doses (5mg/kg) produced sustained serum HZ-0408b levels. The elimination half-life (t_1/2_) of HZ-0408b after administration was up to 13.5 days in monkeys. The PK parameters are summarized in **Table 1** and revealed the maximum serum concentration (C_max_) after HZ-0408b injection ranged from 10.69 μg/mL at a dosage of 1 mg/kg to 23.88 μg/mL in monkeys at a dosage of 5 mg/kg. The corresponding T_max_ was observed at 2–3 days post-dosing, suggesting that the absorption of HZ-0408b was relatively slow from the injection site. Collectively, these data clearly showed that HZ-0408b could be safely administered at doses that can achieve potentially therapeutic serum levels.

**Table 1:** PK parameters of HZ-0408b after single administration.

Dosage	Sex	AUC(0-t)	AUC(0-∞)	t1/2	Tmax	CL	Vz	Cmax
mg/kg		mg/L*h	mg/L*h	d	h	L/h/kg	L/kg	mg/L
1	F	863.58	1020.793	2.7	72	0.001	0.092	6.52
	M	3754.525	3874.355	7.8	8	0	0.07	14.86
5	F	7214.735	7807.16	14.2	72	0.001	0.315	20.22
	M	8422.54	9040.203	12.8	72	0.001	0.245	27.54

AUC(0-∞) = area under the plasma concentration-time curve extrapolated to infinity; AUC(0-t) = area under the plasma concentration-time curve from time zero to the time of the last quantifiable concentration; t1/2 = plasma elimination half-life; Tmax = time when Cmax was reached; Cmax = maximum observed serum concentration; Vz = apparent volume of distribution during terminal elimination phase; CL = serum clearance.

### The Antibody Ameliorated Collagen-Induced Arthritis in Cynomolgus Monkey

**Figure 4:**
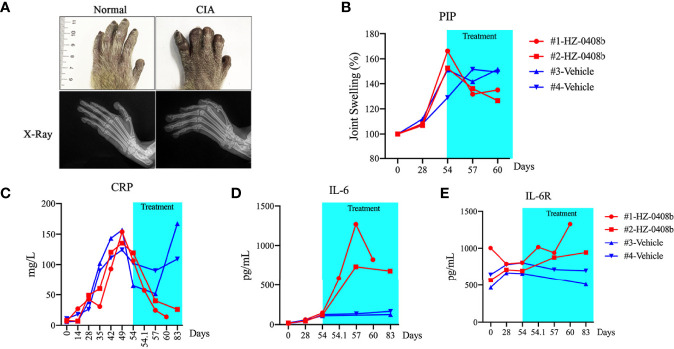
HZ-0408b ameliorates collagen-induced arthritis in cynomolgus monkey. **(A)** Representative pictures of the monkey joint swelling (top) and X-ray (bottom) before HZ-0408b treatment are shown. **(B)** Quantification of swelling of PIP joints in CIA monkeys with HZ-0408b treatment. Each line represents one animal. **(C)** CRP levels in monkeys treated with or without HZ-0408b. Blood was collected from the femoral vein with a syringe containing heparin sodium at indicated time points. CRP level was measured with an automatic analyzer. Plasma IL-6 **(D)** and IL-6R **(E)** levels in CIA monkeys with or without HZ-0408b treatment was measured by ELISA as previously described.

In order to assess the efficacy of the antibody, we next examined whether HZ-0408b can ameliorate arthritis with a monkey collagen-induced arthritis (CIA) model. CIA was induced by immunization of bovine type II collagen in female cynomolgus monkeys (**Figure 4A**). X-ray results demonstrated that the affected joints of CIA monkeys had marginal and central articular erosions with soft tissue swelling and peri-articular osteopenia (**Figure 4A**, bottom). HZ-0408b (30mg/ml) was administrated on Day 54 after the onset of arthritis. We then monitored the swelling of proximal interphalangeal (PIP) joints of fore and hind limbs (without thumb) by measuring the longitudinal and transverse axes of PIP joints and calculated the oval area of each PIP. As shown in **Figure 4B**, joint swelling is 150% of oval area at the time of HZ-0408b injection (Day 54). On day 6 after HZ-0408b treatment (Day 60), we observed a dramatic decrease in joint swelling (130%) (**Figure 4B**).

Increased circulating levels of IL-6 stimulate hepatocytes to produce acute-phase reactants such as C-reactive protein (CRP), a marker of systemic inflammation, fibrinogen, and serum amyloid A (18). We then measured the serum CRP levels. As shown in **Figure 4C**, all animals exhibited elevated CRP levels on day 54 (before HZ-0408b injection). In the control monkey, the CRP level was steady during the administration period. In contrast, in the HZ-0408b-treated monkey, CRP level was remarkably decreased at 1h after injection of HZ-0408b, and the CRP became normal at day 6 after HZ-0408b treatment (**Figure 4C**). These results indicated that HZ-0408b ameliorated collagen-induced arthritis in cynomolgus monkeys.

Blood chemistry showed that the change of mean values of aspartate aminotransferase (AST), alanine aminotransferase (ALT), total cholesterol (TC), lactate dehydrogenase (LDH), and triglyceride (TG) was in the normal range in animals that received HZ-0408b treatment (Data not shown). In all animals, IL-6 was not detectable in plasma before sensitization, and elevated production of IL-6 was observed after the onset of arthritis (Day 54). In control monkey, IL-6 level maintained steady until Day 84. However, in the HZ-0408b-treated monkeys, plasma IL-6 increased remarkably until day 3 after treatment and thereafter decreased gradually until day 6 (**Figure 4D**). IL-6R was detectable in plasma before sensitization in all monkeys. In the control monkey, the IL-6R level remained unchanged from before sensitization through Day 84. In contrast, in the HZ-0408b-treated monkey, the IL-6R concentrations increased from Day 3 until Day 6 after HZ-0408b treatment (**Figure 4E**).

## Discussion

RA is an autoimmune disease characterized by synovial inflammation causing a symmetrical, polyarticular arthritis. It is well established that IL-6 plays an essential role in the pathophysiology of RA. In recent years, remarkable advances have been made in translating the biology of IL-6 to the treatment of patients with various autoimmune diseases, including RA. To date, clinically available anti-IL-6/IL-6R therapies include anti-IL-6 mAb Siltuximab, and anti-IL-6R mAb Tocilizumab and Sarilumab. Tocilizumab has been approved in several countries since 2005 and is currently used to treat RA, giant cell arteritis, and juvenile idiopathic arthritis (10, 11). Sarilumab was approved in the USA for the treatment of refractory RA in 2017 (19). Additionally, Siltuximab, which is a chimeric anti-IL-6 mAb, was approved by FDA for the treatment of patients with iMAD (20). In addition, there are currently three active novel antibodies against IL-6 ligand in the clinical trial stage (12, 21). However, no mAb targeting IL-6 ligand is available clinically for RA treatment so far.

In this study, we developed a novel humanized anti-IL-6 antibody HZ-0408b with the anti-RA therapeutic potential. We demonstrate that 1) HZ-0408b selectively bound to human IL-6 ligand and inhibits IL-6 binding to IL-6R in a dose-dependent manner, 2) HZ-0408b inhibited the synergistic induction of SAA expression by IL-6 and IL-1β in HepG2 cells, and 3) HZ-0408b suppresses the proliferation of DS-1 cells induced by IL-6. These lines of evidence clearly showed that HZ-0408b is a potent selective inhibitor of IL-6 signaling with high species and antigen specificity. We further examined the therapeutic potential of HZ-0408b in a cynomolgus monkey RA model. IL-6 inhibition ameliorated joint swelling, accompanied by remarkably reduced plasma CRP levels when HZ-0408b was administrated after the onset of arthritis in the monkey CIA model. Our data also showed that HZ-0408b outperformed Siltuximab in antigen-binding activity, blocking IL-6 and IL-6R interaction, and neutralizing activity. Moreover, compared to Siltuximab, which is a chimeric human-mouse mAb, HZ-0408b is a humanized mAb, which, in principle, has a minimal immunogenicity and human anti-mouse antibodies (HAMA) response.

IL-6 receptor blockade treatment with the mAb Tocilizumab and Sarilumab represents an important advancement for RA treatment. However, there is no agent against human IL-6 unbound ligands available clinically for RA treatment so far. The preference to target IL-6 receptors rather than IL-6 is partially because the concentrations of the receptors have less interpatient variability than concentrations of IL-6, which may potently simplify does and regimen selection (22, 23). For example, the daily production of IL-6 ligand ranges from several ug/day in most individuals to several mg/day in case of acute infection (24–26). In RA patients, serum IL-6 concentrations are significantly higher, with a mean of 52.7pg/ml compared to 5.1pg/ml in normal (27). However, comparative data from clinical trials for Olokizumab versus Tocilizumb treatment in RA suggest no difference in safety or efficacy between blocking the receptor and the ligand (28). Further investigations are needed to explore the relevance between the efficacy of anti-IL-6 mAb treatment and serum IL-6 levels with an arthritis animal model.

In our monkey CIA model, IL-6 level rapidly increased after HZ-0408b injection and thereafter decreased. This phenomenon, which is consistent with the previous report (17), may result from inhibition of IL-6 binding to IL-6R by HZ-0408b, which prevents its consumption by cells and accumulates more circulating IL-6 in the form of stable monomeric IL-6/HZ-0408b complexes (24). Previous clinical study showed that the administration of Tocilizumab to patients with RA resulted in increased blood levels of both IL-6 ligand and IL-6 receptor (29). Further studies are warranted to investigate the mechanism of IL-6 ligand and IL-6 receptor elevation by anti-IL-6/IL-6R agents administration and its biological consequences.

Of note, we found that HZ-0408b is well tolerated, and no treatment-related toxic effects were observed on a comprehensive list of clinical observations by performing toxicology studies in cynomolgus monkeys. One of the major safety concerns of monoclonal antibody therapy is the development of anti-drug antibodies, which may cause loss of efficacy and/or immune-mediated adverse reactions (30). Further studies evaluating the immunogenicity of HZ-0408b should be performed to test the anti-drug antibody (ADA) responses in subsequent GLP (good laboratory practice) IND (investigational new drug)-enabling pharmacology and toxicology studies in either monotherapy or in combination with DMARDs.

Deregulation of IL-6 production has been shown to play a pivotal role in inflammatory autoimmune diseases like RA, juvenile idiopathic arthritis, adult-onset Still’s disease, giant cell arteritis, and Castleman disease (1, 2). More recently, several studies have reported elevated serum concentrations of IL-6 in severe COVID-19 (31, 32). An agent blocking IL-6 actions could be therapeutically beneficial for these diseases. In this study, we demonstrated that a novel humanized anti-IL-6 mAb HZ-0408b potently binds to IL-6, specifically inhibits IL-6 signaling, and can ameliorate established arthritis in CIA cynomolgus monkeys. Therefore, HZ-0408b — having proved its efficacy and tolerable safety — may serve as a novel therapeutic agent for treating RA, as well as other diseases.

## Data Availability Statement

The original contributions presented in the study are included in the article/**Supplementary Material**. Further inquiries can be directed to the corresponding authors.

## Ethics Statement

The animal study was reviewed and approved by iPhase Pharma Services, Ltd. Institutional Animal Care and Use Committee.

## Author Contributions

FG, HL, and JH contributed to the conception and design of the study. XL, LL, QW, FJ, and PZ performed the experiments and statistical analysis. XL wrote the first draft of the manuscript. LL wrote sections of the manuscript. All authors contributed to manuscript revision, read, and approved the submitted version.

## Funding

This work was supported by the funding from IPHASE Therapeutic and the seed grant from Coriell Institute for Medical Research.

## Conflict of Interest

HL is the founder of IPHASE Therapeutic. LL, QW, FJ, and PZ are employed by IPHASE Therapeutic.

The remaining authors declare that the research was conducted in the absence of any commercial or financial relationships that could be construed as a potential conflict of interest.

The authors declare this study received funding from company IPHASE Therapeutics. The funder had the following involvement in the study: conception and design of the study.

## Publisher’s Note

All claims expressed in this article are solely those of the authors and do not necessarily represent those of their affiliated organizations, or those of the publisher, the editors and the reviewers. Any product that may be evaluated in this article, or claim that may be made by its manufacturer, is not guaranteed or endorsed by the publisher.

## Supplementary Material

Supplementary Figure 1 Cross-reactivity of HZ-0408b to other cytokines and GP130. 96-well plates were coated with indicated cytokines and GP130. Increasing concentrations of HZ-0408b were added and the binding activity of the HZ-0408b was measured by ELISA using an HRP-conjugated anti-HZ-0408b antibody.

Supplementary Figure 2 Immunoblot assay to determine the type of epitope for HZ-0408b. 30ug heat denatured rhIL-6-his was subjected to SDS-PAGE and HZ-0408b was used as primary antibody for immunoblot assay.

Supplementary Figure 3 Pharmacokinetic analysis of HZ-0408b in cynomolgus monkey. Both male (M) and female (F) cynomolgus monkeys were dosed with increasing amount of HZ-0408b (1mg/kg, and 5mg/kg). Blood was collected by venipuncture into tubes with no anticoagulant at indicated time points. Serum level of HZ-0408b was measure by ELISA as described in Methods using IL-6 protein as coating reagent, followed by detection with an HRP-conjugated anti-human IgG antibody.

## Abbreviations

IL-6, Interleukin-6; IL-6R, Interleukin-6 receptor; RA, Rheumatoid arthritis; STAT3, Signal transducer and activator of transcription 3; mAb, Monoclonal antibody; CIA, collagen-induced arthritis; LIF, Leukemia inhibitory factor.
