# Supplementary figures and images for: A Novel Humanized Anti-Interleukin-6 Antibody HZ0408b With Anti-Rheumatoid Arthritis Therapeutic Potential
Source: Front Immunol. 2022 Jan 19;12:816646. doi: 10.3389/fimmu.2021.816646 (PMC8808405; doi:10.3389/fimmu.2021.816646)

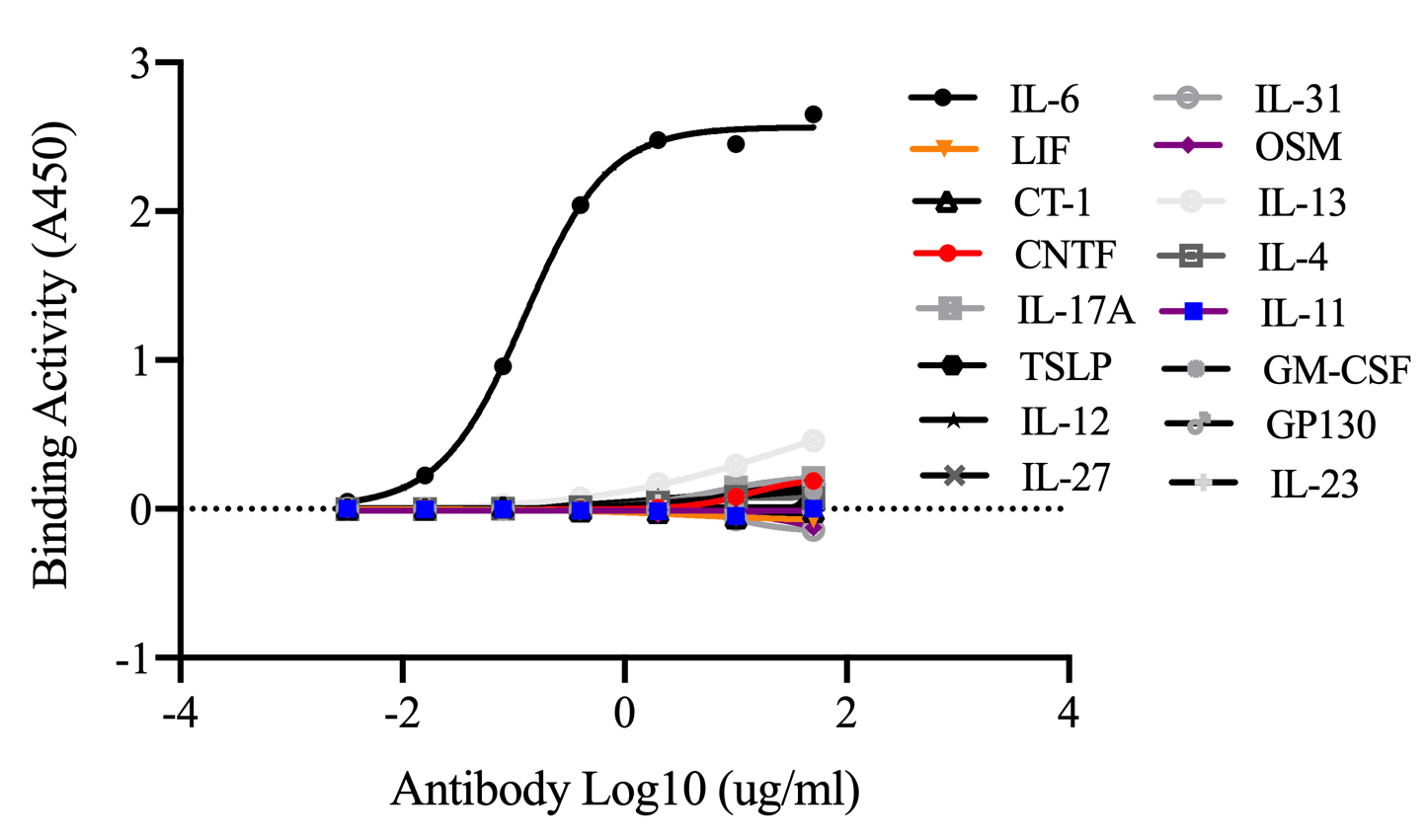

Supplement: Supplementary Figure 1 — Cross-reactivity of HZ-0408b to other cytokines and GP130. 96-well plates were coated with indicated cytokines and GP130. Increasing concentrations of HZ-0408b were added and the binding activity of the HZ-0408b was measured by ELISA using an HRP-conjugated anti-HZ-0408b antibody. [file Image_1.tiff]

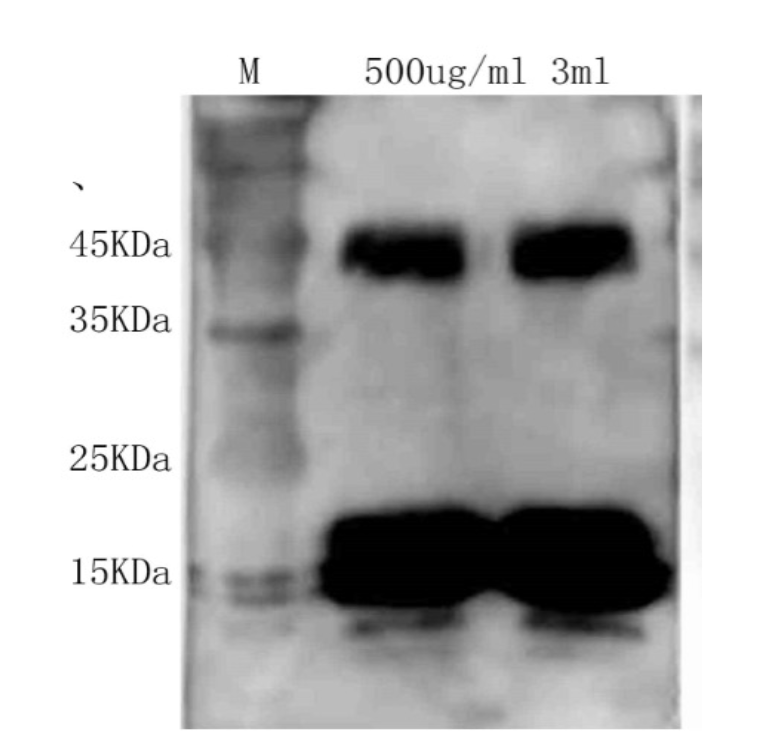

Supplement: Supplementary Figure 2 — Immunoblot assay to determine the type of epitope for HZ-0408b. 30ug heat denatured rhIL-6-his was subjected to SDS-PAGE and HZ-0408b was used as primary antibody for immunoblot assay. [file Image_2.tif]

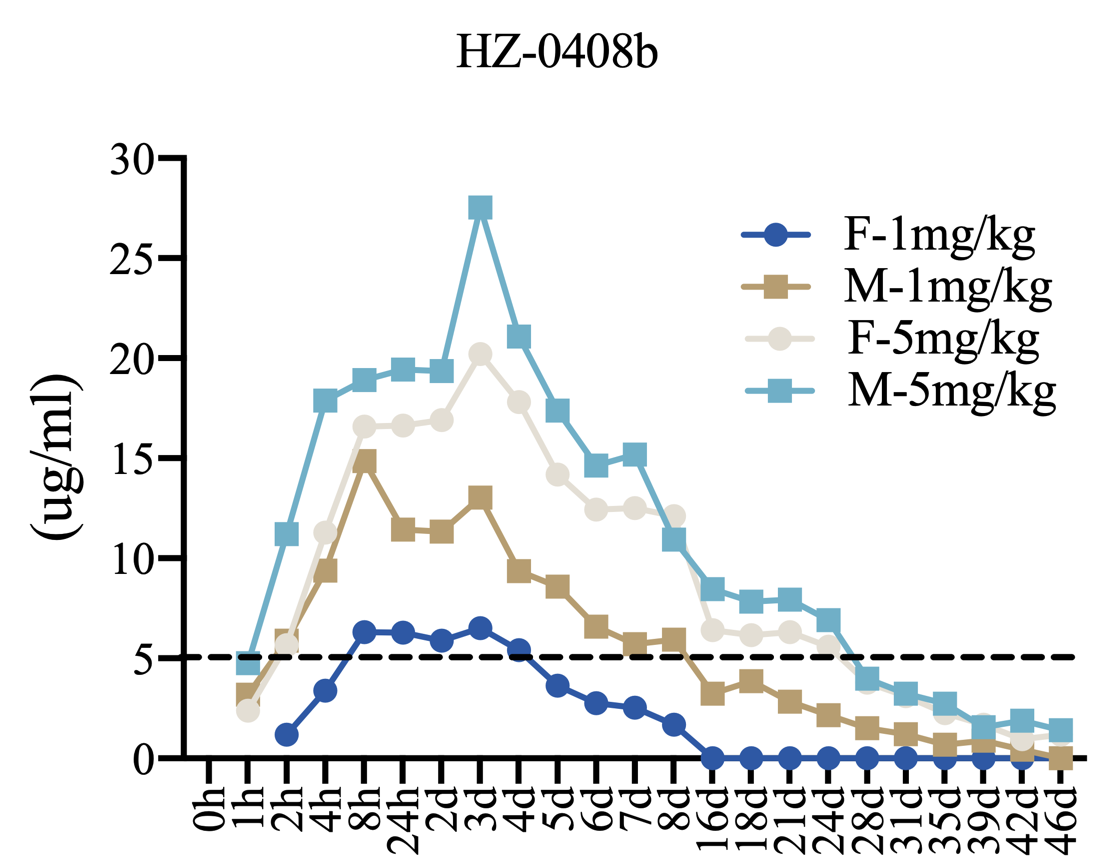

Supplement: Supplementary Figure 3 — Pharmacokinetic analysis of HZ-0408b in cynomolgus monkey. Both male (M) and female (F) cynomolgus monkeys were dosed with increasing amount of HZ-0408b (1mg/kg, and 5mg/kg). Blood was collected by venipuncture into tubes with no anticoagulant at indicated time points. Serum level of HZ-0408b was measure by ELISA as described in Methods using IL-6 protein as coating reagent, followed by detection with an HRP-conjugated anti-human IgG antibody. [file Image_3.tiff]
